# Time, Place, and Partnerships: Event Characteristics and COVID-19 Vaccine Uptake in Community Vaccination Efforts

**DOI:** 10.3390/v18080880

**Published:** 2026-08-11

**Authors:** Shaminul H. Shakib, Seyed M. Karimi, Sirajum Munira Khan, Amen T. Ajamu, Venetia Aranha, Md Yasin Ali Parh, Hamid Zarei, Yuting Chen, Ben Goldman, Dana Novario, Michael Schurfranz, Ciara A. Warren, Trey Allen, Demetra Antimisiaris, Bert B. Little, W. Paul McKinney, Taylor Ingram, Angela J. Graham

**Affiliations:** 1 Department of Public Health, College of Health Sciences, Sam Houston State University, Huntsville, TX 77340, USA; 2 Department of Health Management and Systems Sciences, School of Public Health and Information Sciences, University of Louisville, Louisville, KY 40202, USA; seyed.karimi@louisville.edu (S.M.K.); amen.ajamu@louisville.edu (A.T.A.); hamid.zarei@louisville.edu (H.Z.); demetra.antimisiaris@louisville.edu (D.A.); bert.little@louisville.edu (B.B.L.); 3 Department of Bioinformatics and Biostatistics, School of Public Health and Information Sciences, University of Louisville, Louisville, KY 40202, USA; sirajummunira.khan@louisville.edu; 4 Communicable Disease Surveillance and Education, Louisville Metro Department of Public Health & Wellness, Louisville, KY 40202, USA; venetia.aranha@louisvilleky.gov; 5 Department of Biostatistics, School of Public Health, University of Michigan, Ann Arbor, MI 48109, USA; yasinp@umich.edu; 6 Center for Health Equity, Louisville Metro Department of Public Health & Wellness, Louisville, KY 40202, USA; yuting.chen@louisvilleky.gov (Y.C.); benjamin.goldman@louisvilleky.gov (B.G.); dana.novario@louisvilleky.gov (D.N.); michael.schurfranz@louisvilleky.gov (M.S.); ciara.warren@louisvilleky.gov (C.A.W.); trey.allen@louisvilleky.gov (T.A.); angela.graham@louisvilleky.gov (A.J.G.); 7 Department of Health Promotion and Behavioral Sciences, School of Public Health and Information Sciences, University of Louisville, Louisville, KY 40202, USA; mckinney@louisville.edu; 8 Down Syndrome of Louisville, Louisville, KY 40291, USA; taylori@dsoflou.org

**Keywords:** COVID-19 vaccination, vaccine uptake, community vaccination, local health department, public health practice, vaccine delivery, immunization programs

## Abstract

Understanding how vaccination-event characteristics relate to doses administered can inform public health delivery strategies. This study examined associations between modifiable characteristics of community-based COVID-19 vaccination events and doses administered per event. We conducted a retrospective analysis of 631 vaccination events coordinated by the Louisville Metro Department of Public Health and Wellness (LMPHW) in Jefferson County, Kentucky, between March 2021 and May 2022. The outcome was the number of COVID-19 vaccine doses administered per event. Multivariable regression models assessed associations between event characteristics and doses administered per event. Across all events, 27,767 COVID-19 vaccine doses were administered. Each additional hour of event duration was associated with 21.6% more doses administered per event (95% CI: 14.0–29.8%). Weekend events were associated with 87.8% more doses administered per event than weekday events (95% CI: 45.7–142.0%). Compared with government-hosted events, 44.7% more doses were administered at non-profit organizations, 110.0% more at business organizations, and 57.8% more at medical service providers. LMPHW-only events were associated with 198.4% more doses administered per event than partnered events (95% CI: 120.1–304.6%). Principal findings were generally consistent across sensitivity analyses. These findings may help inform planning of future community vaccination and other immunization initiatives.

## 1. Introduction

The COVID-19 pandemic highlighted longstanding inequities in healthcare access in the United States, including disparities in COVID-19 vaccination coverage across racial, ethnic, and socioeconomic groups [1,2,3,4,5]. In response, local health departments and community organizations implemented a wide range of community-based vaccination strategies, including mobile clinics, temporary vaccination sites, and outreach events designed to improve access [6,7,8,9]. Although substantial research has examined demographic disparities in vaccination, less attention has been given to how vaccination services themselves are organized and delivered. Understanding how operational characteristics of community vaccination events, including when and where services are offered and which organizations are involved, are associated with event-level vaccine delivery provides practical evidence to inform future public health vaccination efforts.

Community-based vaccination strategies have been widely promoted to increase access and reduce disparities [6,7,8,9]. However, the role of modifiable event characteristics, such as event timing, hosting organization type, and partnership structure, in relation to the number of vaccine doses administered during community vaccination events remains understudied. Prior research has largely focused on population-level disparities in vaccination coverage [2,3,4,5], with less attention to how the design and implementation of vaccination efforts are associated with event-level vaccination delivery in real-world community settings [9]. Understanding these operational factors may help inform the planning and implementation of community vaccination programs in real-world community settings.

Despite widespread implementation of community vaccination programs during the COVID-19 pandemic, relatively little empirical evidence is available to guide operational decisions regarding community vaccination event design. Local health departments routinely determine when vaccination events should occur, how long they should operate, where they should be located, and whether they should be conducted independently or in partnership with community organizations. These operational decisions have important implications for resource allocation and service delivery, yet their association with the number of vaccine doses administered per event has received comparatively little attention. Although COVID-19 vaccination remains an important strategy for reducing severe disease and mortality [10,11], understanding how modifiable features of vaccination delivery relate to doses administered may also inform planning for future vaccination campaigns and other community-based preventive health initiatives.

This study evaluated associations between modifiable characteristics of community-based COVID-19 vaccination events and the number of vaccine doses administered during those events. Specifically, we examined event duration, timing (time of day and day of week), season, hosting organization type, and partnership status among 631 community vaccination events coordinated by the Louisville Metro Department of Public Health and Wellness (LMPHW) in Jefferson County, Kentucky, from March 2021 through to May 2022. By examining operational characteristics associated with doses administered per event, this study provides practical evidence to inform the planning and implementation of future community vaccination programs and other community-based immunization efforts.

## 2. Methods

### 2.1. Study Design and Setting

A retrospective observational study of community-based COVID-19 vaccination events coordinated by the LMPHW in Jefferson County, Kentucky, was conducted. Events were held between March 2021 and May 2022 as part of LMPHW’s vaccination campaign, implemented with local organizational partners. Vaccines were administered free of charge. Each observation represented a single vaccination event on a specific date and of a specified duration. All community-based vaccination events coordinated by LMPHW during the study period were eligible for inclusion. Events without recorded dose counts were excluded from the analytic sample because the primary outcome could not be determined.

### 2.2. Data Sources

Event-level data were derived from routine programmatic records maintained by LMPHW and included information on event timing, duration, day of the week, hosting organization, and partnership status. Partner organizations assisted with logistics, workforce support, and other operational duties. The study outcome was measured as the number of COVID-19 vaccine doses administered to Jefferson County residents at each event. A total of 662 events were identified during the study period; 31 events with missing dose counts were excluded, resulting in an analytic sample of 631 events.

### 2.3. Measures

The primary outcome was the total number of COVID-19 vaccine doses administered during each community vaccination event. Events with unknown hosting organization information were retained and analyzed as a separate category to preserve the analytic sample.

Independent variables reflected modifiable characteristics of vaccination events. Event duration was measured in hours. An event’s timing was categorized according to its scheduled end time: morning (8:00 am–10:59 am), noon (11:00 am–12:59 pm), afternoon (1:00 pm–4:59 pm), or evening (5:00 pm–9:00 pm). Scheduled end time was used, together with event duration, to distinguish events that extended into later parts of the day. Each observation was classified as a weekday (Monday–Friday) or weekend (Saturday–Sunday) event. Season was coded as spring (March–May), summer (June–August), fall (September–November), or winter (December–February).

Hosting organizations primarily provided space for vaccination events. Each organization was classified into one mutually exclusive category based on its primary organizational function: business organization, non-profit organization, government agency, medical service provider, festival, homeless encampment site, independent organization, inmate transitional service provider, mental health service provider, senior care service provider, or youth service provider (Table S1). Because several hosting categories included relatively few events, mental health service providers were combined with medical service providers, while festivals, homeless encampment sites, independent organizations, inmate transitional service providers, senior care service providers, and youth service providers were combined into a single “Other service provider” category for analysis (Table S2). Organization names were reviewed by multiple investigators, and each organization was assigned to one mutually exclusive category based on the organization type recorded in programmatic records and, when needed, the organization’s self-described primary function. Classification decisions were reviewed by the investigative team and resolved by consensus. Hosting organization information was missing for 87 of the 662 identified events. One of these events also lacked a dose count and was excluded, leaving 86 events classified as “Unknown” in the analytic sample.

Partnership status captured whether events were conducted solely by the LMPHW or in collaboration with at least one partner organization. Partner organizations assisted with event setup, logistics, and vaccine administration through skilled nurses or trained health care workers. For a subset of events (n = 36), records indicated collaboration but did not identify the partner organization; these events were retained and classified as partnered events (Table S3).

### 2.4. Statistical Analysis

Descriptive statistics were calculated for all study variables. Frequencies and percentages were reported for categorical variables, whereas continuous variables were summarized using means and standard deviations (SDs). Figures were generated to display monthly trends in vaccine doses administered and event counts by event timing, hosting organization, partnership status, and day of week.

Because the distribution of vaccine doses administered per event was highly right-skewed, the primary outcome was transformed using the natural logarithm of the observed dose count [ln(doses)] before regression analyses. Events with zero administered doses were excluded from the log-transformed analyses because the natural logarithm of zero is undefined. Unadjusted and adjusted linear regression models were estimated to assess associations between event characteristics and the log-transformed number of COVID-19 vaccine doses administered per event. The adjusted model included event duration, time of day, day of week, season, hosting organization type, and partnership status. Sensitivity analyses included a linear regression model using the untransformed dose count and an adjusted negative binomial regression model using the same untransformed outcome and covariates as the primary model. These analyses evaluated whether the direction, magnitude, and statistical significance of the observed associations were consistent across alternative outcome and model specifications. The primary adjusted model included 623 events with positive dose counts and complete covariate data, whereas both adjusted sensitivity analyses included 630 events with complete covariate data.

For models with log-transformed outcomes, exponentiated regression coefficients were used to estimate relative differences in the number of COVID-19 vaccine doses administered per event. Percentage differences were calculated as 100 × [exp(b) − 1], with corresponding 95% confidence intervals calculated using the same transformation.

Statistical significance was defined as a two-sided *p*-value < 0.05. Data were managed in KNIME Analytics Platform, version 5.1 (KNIME AG, Zurich, Switzerland), and analyzed in Stata, version 18 (StataCorp LLC, College Station, TX, USA). This study used de-identified programmatic data, was approved as exempt by the University of Louisville (IRB #22.0681) and the Kentucky Cabinet for Health and Family Services (IRB #CHFS-IRB-DPH-FY22-33), and did not require informed consent. The study adhered to the Strengthening the Reporting of Observational Studies in Epidemiology (STROBE) guidelines [12].

## 3. Results

### 3.1. Descriptive Statistics

A total of 27,767 COVID-19 vaccine doses were administered across 631 vaccination events in Jefferson County, Kentucky, between March 2021 and May 2022, with a mean event duration of 3.6 h (Table 1).

Most events were scheduled in the afternoon (n = 343; 54.4%), followed by noon (n = 131; 20.8%), evening (n = 112; 17.8%), and morning (n = 45; 7.1%). Afternoon events accounted for the largest share of all doses administered (n = 19,393; 69.8%), whereas morning events accounted for the smallest share (n = 840; 3.0%). Most events were held on weekdays (n = 536; 84.9%). Although weekend events accounted for 15.1% of events (n = 95), they contributed 30.1% of doses administered (n = 8349).

Event distribution varied by season. The number of events increased from spring 2021 (n = 99; 15.7%) to summer (n = 203; 32.2%) and fall 2021 (n = 189; 29.9%), and then declined in winter 2021–2022 (n = 85; 13.5%) and spring 2022 (n = 55; 8.7%). Mean doses per event were higher in winter 2021–2022 (72.8; SD = 120.9) and spring 2022 (83.9; SD = 75.2) than in earlier seasons.

Hosting organizations were most often non-profit organizations (n = 233; 36.9%), followed by government agencies (n = 133; 21.1%), business organizations (n = 82; 13.0%), medical service providers (n = 55; 8.7%), and other service providers (n = 42; 6.7%). Events with unknown host type accounted for 13.6% of events (n = 86). Mean doses per event were highest for government agency-hosted events (57.0; SD = 65.8) and non-profit organizations (53.4; SD = 84.4), and lowest for other service providers (21.3; SD = 25.8).

Partnered events accounted for 89.5% of all events (n = 565), whereas LMPHW-only events accounted for 10.5% (n = 66). Mean doses per event were more than twice as high for LMPHW-only events (89.3; SD = 69.4) as for partnered events (38.7; SD = 62.0).

A descriptive comparison indicated that events excluded because of missing dose counts tended to be shorter, were more frequently conducted during winter 2021–2022, and were all partnered events (Table S6).

**Figure 1:**
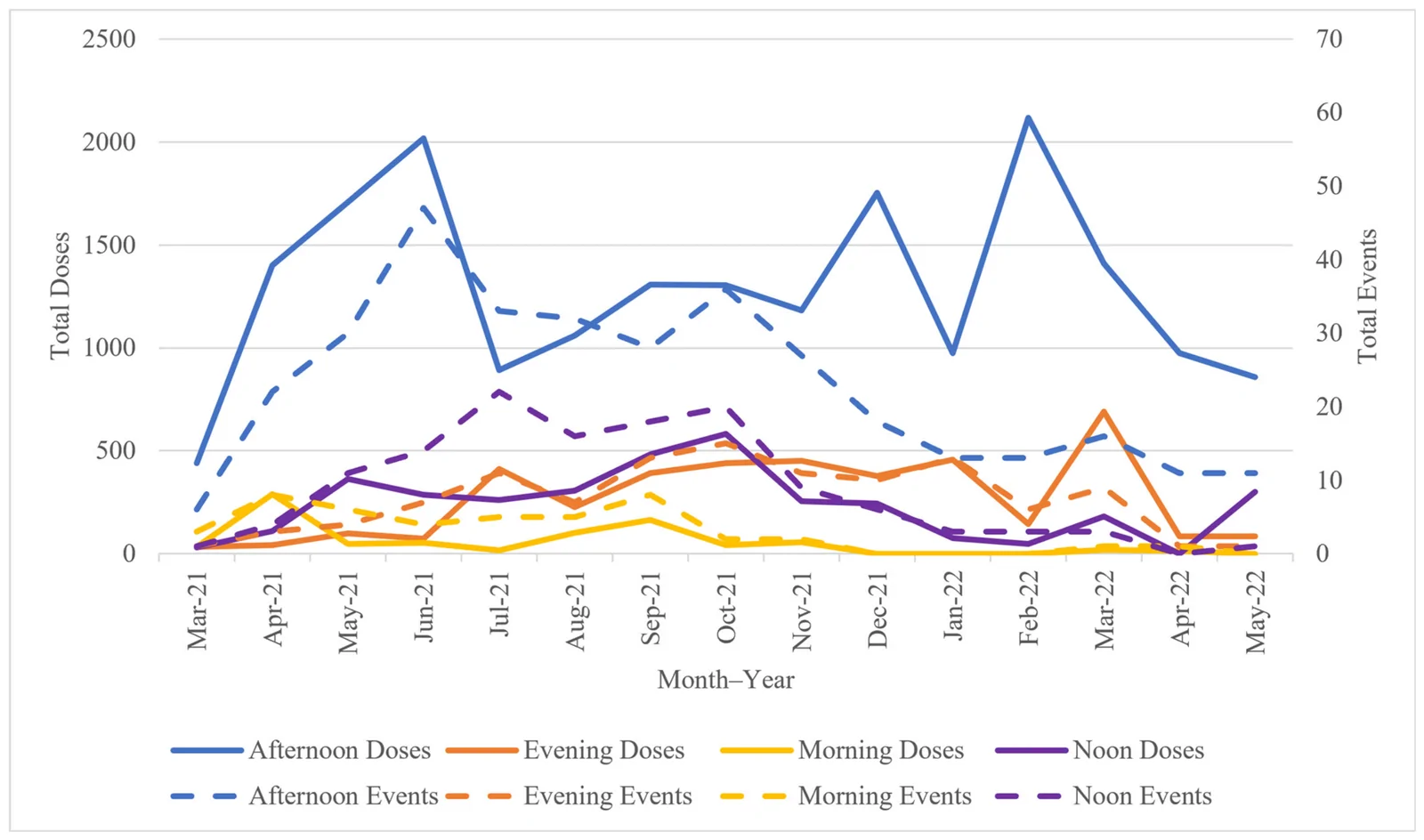
Monthly COVID-19 Vaccine Doses Administered and Events by Event Time—LMPHW, Jefferson County, KY, Mar 2021–May 2022. Note. Monthly vaccine doses administered (solid lines; left y-axis) and number of events (dashed lines; right y-axis) by event time, Louisville Metro Department of Public Health and Wellness (LMPHW) vaccine campaign—Jefferson County, Kentucky, March 2021–May 2022. Doses reflect events with non-missing dose counts; event counts include all scheduled events.

Monthly trends by event time indicated that afternoon events accounted for the largest share of events and doses, whereas morning events were infrequent and contributed minimally to total doses (Figure 1).

**Figure 2:**
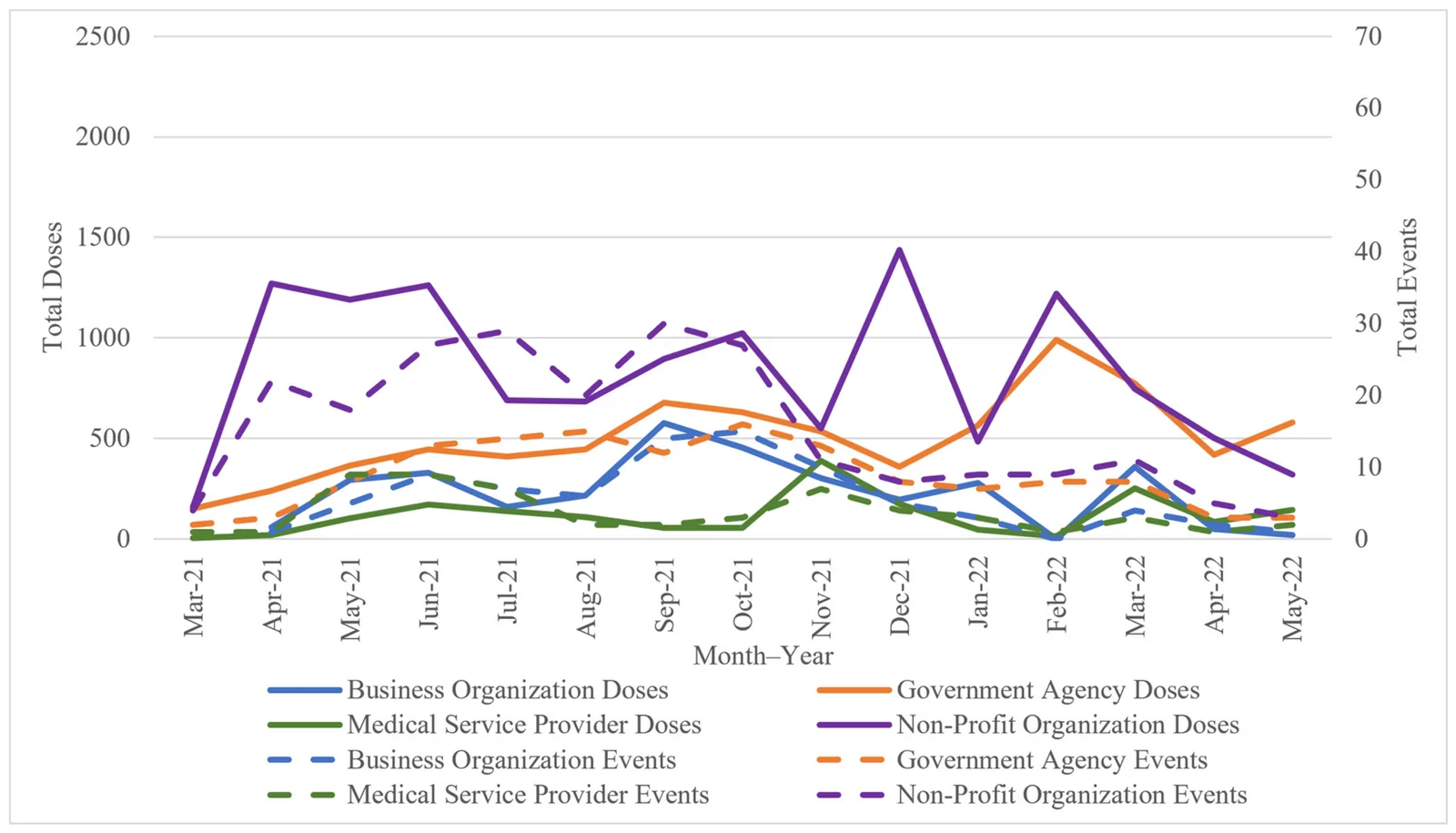
Monthly COVID-19 Vaccine Doses Administered and Events by Hosting Organization Type—LMPHW, Jefferson County, KY, Mar 2021–May 2022. Note. Monthly vaccine doses administered (solid lines; left y-axis) and number of events (dashed lines; right y-axis) by hosting organization type, LMPHW vaccine campaign—Jefferson County, Kentucky, March 2021–May 2022. Doses reflect events with non-missing dose counts; event counts include all scheduled events.

Monthly trends by hosting organization type indicated that non-profit and government-hosted events accounted for the largest share of events and doses over time (Figure 2).

Partnered events comprised most events each month, whereas LMPHW-only events, although fewer, generally had more doses administered per event (Figure S1). Weekday events accounted for most events, but weekend events contributed disproportionately to total doses (Figure S2).

### 3.2. Regression Analysis

In adjusted models, each additional hour of event duration was associated with 21.6% more doses administered per event (95% CI: 14.0–29.8%) (Table 2). Compared with afternoon events, noon events were associated with 26.2% fewer doses administered per event (95% CI: 8.3–40.7% fewer). Morning and evening events did not differ significantly from afternoon events. Weekend events were associated with 87.8% more doses administered per event than weekday events (95% CI: 45.7–142.0%).

Relative to spring 2021, summer 2021 was associated with 50.3% fewer doses administered per event (95% CI: 36.4–61.2% fewer), and fall 2021 was associated with 32.2% fewer doses administered per event (95% CI: 12.3–47.7% fewer). Spring 2022 was associated with 85.5% more doses administered per event than spring 2021 (95% CI: 32.9–159.0%). Winter 2021–2022 did not differ significantly from spring 2021.

Hosting organization type was significantly associated with doses administered per event. Compared with government-hosted events, non-profit-hosted events were associated with 44.7% more doses administered per event (95% CI: 10.5–89.5%), business-hosted events with 110.0% more (95% CI: 54.6–185.4%), and medical service provider-hosted events with 57.8% more (95% CI: 11.0–124.3%). Events without a recorded hosting organization were associated with 58.5% fewer doses administered per event than government-hosted events (95% CI: 40.1–71.3% fewer). LMPHW-only events were associated with 198.4% more doses administered per event than partnered events (95% CI: 120.1–304.6%).

### 3.3. Sensitivity Analysis

Results from models using the untransformed outcome were largely consistent with the primary analysis (Table S4). In the linear model using the untransformed outcome, evening events were associated with 13.3 fewer COVID-19 vaccine doses administered per event (95% CI: −25.9 to −0.7), and winter 2021–2022 was associated with 28.4 more doses administered per event than spring 2021 (95% CI: 12.2 to 44.7). The additional negative binomial sensitivity analysis also supported the principal findings for event duration, weekend scheduling, season, hosting organization type, and partnership status (Table S5). However, associations for time of day varied across model specifications: morning events were associated with fewer doses than afternoon events in the negative binomial model, whereas noon events were not significantly different. Overall, the principal associations were robust, although time-of-day findings should be interpreted cautiously.

## 4. Discussion

The effectiveness of local vaccination campaigns depends not only on vaccine availability but also on how services are delivered [1]. In this evaluation of community-based COVID-19 vaccination events coordinated by the Louisville Metro Department of Public Health and Wellness (LMPHW) in Jefferson County, event characteristics, including duration, scheduling, hosting organization, and partnership structure, were associated with differences in the number of COVID-19 vaccine doses administered per event. These results suggest that decisions about when, where, and how vaccination services are offered may be relevant to differences in the number of doses administered during community vaccination events across diverse public health settings. Together, this study adds to growing evidence that service delivery design is an important component of effective vaccination strategies and public health practice. These findings may help inform the planning and implementation of community-based vaccination strategies during future infectious disease outbreaks and other public health emergencies.

Longer event duration and weekend scheduling were associated with more COVID-19 vaccine doses administered per event, suggesting that extending service availability and offering vaccination opportunities outside the traditional workweek may expand opportunities for community vaccination. Compared with afternoon events, noon events were associated with fewer doses administered per event, whereas morning and evening events did not differ significantly after adjustment. These findings are consistent with previous research emphasizing that community-based and mobile vaccination strategies can reduce structural barriers by offering services at times and locations that better align with community needs [8,13]. Together, these findings suggest that operational decisions regarding event timing and duration may inform community vaccination delivery.

Differences in the number of COVID-19 vaccine doses administered per event by hosting organization type suggest that where vaccination services are delivered may reflect differences in how communities interact with vaccination settings. Compared with government-hosted events, those hosted by non-profit organizations, businesses, and medical service providers were associated with more doses administered per event, even after adjustment for other measured event characteristics. These differences may reflect variation in event settings, accessibility, and the populations reached across community venues. Previous studies of community-based vaccination efforts have similarly shown that delivering services in accessible, community-embedded settings is associated with improved participation [6,14]. Together, these findings suggest that venue selection may be an important operational consideration when planning community-based vaccination efforts.

The association between partnership status and the number of COVID-19 vaccine doses administered per event warrants careful interpretation. LMPHW-only events were associated with more COVID-19 vaccine doses administered per event than partnered events; however, doses administered per event may not fully capture the broader objectives of partnership-based approaches, including outreach and equity-focused engagement. Partnered events may, in some cases, be intended to reach populations facing greater barriers to vaccination, where participation per event may be lower but the event’s public health value may not be reflected by dose count alone. Some partnered events may have been designed around outreach or equity-related objectives rather than maximizing event-level dose counts. In addition, this study did not capture variation in partner type, roles, or level of engagement, all of which may influence the effectiveness of collaborative vaccination efforts. Future research should examine how partnership characteristics relate to both vaccination reach and equity outcomes.

Seasonal variation in the number of COVID-19 vaccine doses administered per event was also observed, with fewer doses administered during summer and fall 2021 and more doses administered during spring 2022 relative to spring 2021. These differences likely reflect the evolving stage of the COVID-19 vaccination campaign rather than seasonal effects alone, including changes in vaccine demand, eligibility recommendations, perceived risk, and public health priorities over time. Although this study was not designed to evaluate these factors directly, the findings suggest that temporal context should be considered alongside event characteristics when planning community vaccination strategies and outreach efforts.

This study did not include individual-level demographic data. However, the findings identify modifiable event characteristics that may be considered when planning community-based vaccination efforts. Health departments may consider offering longer-duration events and increasing weekend availability to expand opportunities for vaccination. Aligning event timing with community needs and utilizing community-based venues, such as non-profit organizations, business organizations, and medical service providers, may be considered when seeking to increase the number of doses administered during community vaccination events. Partnerships remain an important strategy, but their effectiveness may depend on how they are structured and implemented. Taken together, these findings suggest that operational decisions regarding event design may inform community vaccination delivery and broader preventive service delivery efforts.

## 5. Limitations

This study has several limitations. First, the outcome was measured as the number of COVID-19 vaccine doses administered per event rather than individual-level vaccination status, precluding assessment of vaccination uptake across demographic groups or identification of the populations reached by different event types. In addition, the observational design limits causal inference. Second, the absence of population denominators limited the ability to estimate vaccination coverage; higher numbers of doses administered per event may reflect both increased demand and improved access. Third, unmeasured factors, including outreach activities, local COVID-19 transmission patterns, community demand, and variation in partnership structure or intensity, may have influenced the number of doses administered per event. Measurement error in administrative records and residual confounding may also have introduced bias. These factors may have led to either overestimation or underestimation of associations, and the direction and magnitude of these potential biases could not be determined. Fourth, season was a relatively coarse measure of calendar time and may not have fully captured time-varying changes in vaccine eligibility, booster recommendations, circulating variants, public messaging, and community demand across the study period; residual temporal confounding may therefore remain. Fifth, 31 events were excluded because dose counts were missing. These events were shorter on average, were concentrated in winter 2021–2022, and were all partnered events; therefore, selection bias related to missing outcome data cannot be ruled out. Sixth, information on vaccine type and dose sequence was not available. Finally, findings are based on a single county’s vaccination program and may not be generalizable to other settings with different population characteristics or health system structures.

## 6. Conclusions

In this evaluation of community-based COVID-19 vaccination events coordinated by a local health department, event characteristics, including duration, scheduling, hosting organization, and partnership structure, were associated with differences in the number of COVID-19 vaccine doses administered per event. These findings suggest that operational decisions regarding service delivery are relevant to differences in the number of doses administered during community vaccination events. Designing vaccination efforts that align with community needs and service delivery contexts may strengthen ongoing COVID-19 vaccination efforts and help inform the planning of future vaccination programs and other community-based preventive service delivery initiatives.

## Figures and Tables

**Table 1:** Characteristics of COVID-19 Vaccination Events and Doses Administered During the LMPHW Vaccination Campaign—Jefferson County, KY, March 2021–May 2022.

Characteristic	Events: n (%)	Vaccines: n (%)	Vaccines: Mean (SD)
Total	631 (100)	27,767 (100)	44.0 (64.6)
Mean (SD) event duration (hours)	3.6 (2.0)		
Event Time			
Morning	45 (7.1)	840 (3.0)	18.7 (17.2)
Noon	131 (20.8)	3523 (12.7)	26.9 (35.9)
Afternoon	343 (54.4)	19,393 (69.8)	56.5 (79.5)
Evening	112 (17.8)	4011 (14.4)	35.8 (37.9)
Day of Week			
Weekday	536 (84.9)	19,418 (69.9)	36.2 (45.4)
Weekend	95 (15.1)	8349 (30.1)	87.9 (118.1)
Season			
Spring 2021	99 (15.7)	4600 (16.6)	46.5 (56.0)
Summer 2021	203 (32.2)	5707 (20.6)	28.1 (39.9)
Fall 2021	189 (29.9)	6657 (23.9)	35.2 (36.1)
Winter 2021–2022	85 (13.5)	6189 (22.3)	72.8 (120.9)
Spring 2022	55 (8.7)	4614 (16.6)	83.9 (75.2)
Event Hosting Organization Type			
Non-Profit Organization	233 (36.9)	12,434 (44.8)	53.4 (84.4)
Government Agency	133 (21.1)	7584 (27.3)	57.0 (65.8)
Business Organization	82 (13.0)	3297 (11.9)	40.2 (46.0)
Medical Service Provider	55 (8.7)	1762 (6.3)	32.0 (31.2)
Other Service Provider	42 (6.7)	895 (3.2)	21.3 (25.8)
Unknown	86 (13.6)	1795 (6.5)	20.9 (19.9)
Event Partnership Status			
Partnered Event	565 (89.5)	21,872 (78.8)	38.7 (62.0)
LMPHW-only Event	66 (10.5)	5895 (21.2)	89.3 (69.4)

Note: Vaccine doses administered per event were highly right-skewed (median [IQR]: 22 (10–50)). Unknown refers to events with no recorded hosting organization. LMPHW-only events were conducted independently by the Louisville Metro Department of Public Health and Wellness without formal external partners. Table values reflect the 631 events with non-missing dose counts. Percentages are column percentages within Events or Vaccines. Mean (SD) reflects the average number of doses administered per event.

**Table 2:** Event Characteristics and Log-Transformed COVID-19 Vaccine Doses Administered per Event During LMPHW Vaccine Campaign—Jefferson County, KY, March 2021–May 2022.

Variable	Unadjusted b (95% CI)	*p* Value	Adjusted b (95% CI)	*p* Value
Duration of Event (per hour)	0.069 (0.024, 0.114)	0.003	0.196 (0.131, 0.261)	<0.001
Event Time (ref: Afternoon)				
Morning	−0.791 (−1.151, −0.431)	<0.001	−0.331 (−0.668, 0.006)	0.054
Noon	−0.707 (−0.938, −0.476)	<0.001	−0.304 (−0.522, −0.087)	0.006
Evening	−0.288 (−0.536, −0.040)	0.023	−0.100 (−0.341, 0.142)	0.418
Weekend (ref: Weekday)	0.592 (0.336, 0.848)	<0.001	0.630 (0.377, 0.884)	<0.001
Season (ref: Spring 2021)				
Summer 2021	−0.610 (−0.882, −0.339)	<0.001	−0.699 (−0.946, −0.452)	<0.001
Fall 2021	−0.259 (−0.533, 0.015)	0.064	−0.389 (−0.647, −0.131)	0.003
Winter 2021–2022	0.145 (−0.183, 0.474)	0.385	0.074 (−0.233, 0.381)	0.636
Spring 2022	0.680 (0.308, 1.051)	<0.001	0.618 (0.284, 0.952)	<0.001
Event Hosting Organization Type (ref: Government Agency)				
Non-Profit Organization	−0.096 (−0.348, 0.156)	0.455	0.370 (0.100, 0.639)	0.007
Business Organization	0.019 (−0.306, 0.344)	0.907	0.742 (0.435, 1.049)	<0.001
Medical Service Provider	−0.341 (−0.710, 0.028)	0.070	0.456 (0.105, 0.808)	0.011
Other Service Provider	−0.741 (−1.148, −0.333)	<0.001	−0.050 (−0.428, 0.329)	0.797
Unknown	−0.449 (−0.769, −0.130)	0.006	−0.880 (−1.248, −0.512)	<0.001
Event Partnership Status (ref: Partnered Event)				
LMPHW-only Event	1.085 (0.794, 1.375)	<0.001	1.093 (0.789, 1.398)	<0.001

Note: b = unstandardized regression coefficient; CI = confidence interval. Estimates are from unadjusted and adjusted linear regression models of the natural logarithm of COVID-19 vaccine doses administered per event. Regression coefficients are presented on the log scale; percentage differences reported in the text were calculated as 100 × [exp(b) − 1]. Sample sizes varied slightly across unadjusted models because of missing covariate data. The adjusted model included 623 events with positive dose counts and complete data on all model covariates. Unknown refers to events with no recorded hosting organization. LMPHW-only events were conducted independently by the Louisville Metro Department of Public Health and Wellness without formal external partners. Reference categories are indicated in parentheses.

## Data Availability

The event-level data are not publicly available because they contain dates, locations, organization identifiers, and programmatic information subject to data-use and privacy restrictions. Requests for access may be directed to the Kentucky Department of Public Health and are subject to agency approval.

## Supplementary Materials

The following supporting information can be downloaded at https://www.mdpi.com/article/10.3390/v18080880/s1. Table S1: List of Hosting Organization Names and Assigned Types; Table S2: Original Hosting Organization Categories and Recoding into the Analytic Categories; Table S3: Collaborators that partnered with LMPHW for Vaccine Events; Table S4: Event Characteristics and COVID-19 Vaccine Doses Administered per Event During LMPHW Vaccine Campaign—Jefferson County, KY, March 2021–May 2022; Table S5: Negative Binomial Sensitivity Analysis of Event Characteristics and COVID-19 Vaccine Doses Administered per Event During the LMPHW Vaccination Campaign, Jefferson County, Kentucky, March 2021–May 2022; Table S6: Comparison of Included Events and Events Excluded Because of Missing Dose Counts; Figure S1: Monthly COVID-19 Vaccine Doses Administered and Events by Partnership Status—LMPHW Vaccine Campaign, Jefferson County, KY, Mar 2021–May 2022; Figure S2: Monthly COVID-19 Vaccine Doses Administered and Events by Day of Week—LMPHW Vaccine Campaign, Jefferson County, KY, Mar 2021–May 2022.

## Abbreviations

The following abbreviation is used in this manuscript:

LMPHW	Louisville Metro Department of Public Health and Wellness
