# Supplementary material for: Time, Place, and Partnerships: Event Characteristics and COVID-19 Vaccine Uptake in Community Vaccination Efforts
Source: Viruses. 2026 Aug 11;18(8):880. doi: 10.3390/v18080880 (PMC13517676; doi:10.3390/v18080880)
Supplement: Supplementary file 1 [file viruses-18-00880-s001.zip › viruses-4405436-supplementary.pdf]

Supplementary Material for: **Time, Place, and Partnerships: Event Characteristics and COVID-19 Vaccine Uptake in Community Vaccination Efforts**

Table S1. List of Hosting Organization Names and Assigned Types

Assigned Organization Type	Hosting Organization Name
Business Organization	KFC
	Oxmoor Lodge Independent Senior Living
	Koch Filter
	The Galt House Hotel
	Praxis of Louisville by Landmark Recovery
	Kaleidoscope Inc
	Teasdale Latin Foods
	Simmons College of Kentucky
	Grade A Auto Parts
	Healthy Trucking of America
	Repak Recycling, LLC
	GlowTouch LLC
	Hazelnut Farm Events
	McDonald's
	Phillip Patterson Painting
	Gathering Club
	Arvato USA - LVL2
	Wild Accelerator
	Galen College of Nursing
	Boone's Gas Station
	Unipak LLC
	Paul Mitchell The School
	Medrano
	Rev-A-Shelf
	Universal Linen
	Comoto Inc. Distribution Center
	Techstyle Fashion Group
	National Tobacco Company
	Kentucky Trailer
	Kentucky Kingdom Theme & Water Park
	Paradise Tomato Kitchens
	Premier Packaging
	Ferrara Candy Company
	Sysco Louisville - Food Distributor & Restaurant Supplies
	Hotel Louisville
	Lantech
Festival	Mayor's Hike, Bike & Paddle
	Olmsted Parks Beer Garden, Seneca Park
	Annual Unity Festival
Governmental Agency	Louisville Metro Department of Corrections
	Louisville Metro Department of Public Works
	South Jefferson Neighborhood Place

Assigned Organization Type	Hosting Organization Name
	Shawnee Community Center
	Dixie Health Center
	Victory Park
	LMG Employees Booster Clinic
	LMPHW Harm Reduction Outreach Services (Syringe Services Program)
	Louisville Metro Emergency Management Services
	Transit Authority of River City (TARC)
	Jefferson Community & Technical College
	Louisville MSD
	Jefferson County Public Schools
	Western Library
	Parkhill Community Center
	Louisville Metro Housing Authority
	Olmstead Middle School
	Jefferson County Judicial Center
	Louisville Fire Training Academy
	Louisville Metro Police
	Iroquois High School
	Shawnee Library
	California Neighborhood Center
	Jefferson County Attorney Child Support Division
	Louisville Metro Public Health and Wellness Department (LMPHW)
	Louisville Free Public Library Main Branch
	Toonerville Trolley Park
	Louisville Zoo
Homeless Encampment Site	Encampment-Jackson
	Homeless camp
	Homeless Encampment
	Mercer Encampment
Independent Organization	West Louisville Urban Coalition
	Black Lives Matter Louisville
	Hope Wellness Center
Inmate Transitional Service Provider	Community Transitional Services (CTS)
Medical Service Provider	Innovative Renal Care University of Louisville Dialysis Center
	American Kidney Center
	Smoketown Family Wellness Center
	Landmark Recovery
	Fresenius Kidney Care
	BMA East Louisville
	VOA Recovery Shelby Men's Recovery Center
	BrightStar Care
	UofL Trager Institute/Republic Bank Foundation Optimal Aging Clinic
	NeuroRestorative

Assigned Organization Type	Hosting Organization Name
	Kentuckiana Nursing Service
Mental Health Service Provider	BHG Louisville Treatment Center
	Wellspring
Non-Profit Organization	Louisville Youth Group Inc, First Lutheran Church
	Church of the Promise
	Speed Art Museum
	YMCA
	Family Community Clinic
	Bethany United Church of Christ
	Neighborhood House
	Americana Center
	Hip Hop N 2 learning
	Cable Baptist Church
	The Healing Place
	UP for Women and Children
	Volunteers of America (VOA)
	Historic Calvary Missionary Baptist Church
	LRCC, The Hope Bus, Change Today, Change Tomorrow
	Beechmont Presbyterian Church
	St. Peter the Apostle Catholic Church
	The Center for Women and Families
	Nicole 50/50 Mentoring Collaborative
	Oxford Houses
	Talbot House
	Kentucky Derby Festival
	St. John Paul II Parish
	HIRA Institute
	Americana World Community Center
	Family Health Centers - Iroquois
	West Chestnut St Baptist Church
	Newburg Church of Christ
	Recovery on Chestnut
	Genesis United Methodist Church
	Ralph Avenue Baptist Church
	Wayside Christian Mission
	Highland Park Missionary Baptist Church
	Village Community Center Inc
	Wesley House Community Services
	St. John Center
	National Association for the Advancement of Colored People (NAACP)
	Collective Hope Louisville
	Louisville Urban League
	Catholic Enrichment Center
	Bates CDC

Assigned Organization Type	Hosting Organization Name
	Louisville Pride Foundation
	Coke Memorial United Methodist Church
	Free2Hope
	Change Today, Change Tomorrow
	Center for Accessible Living
	Harshaw Family Foundation Animal Shelter
	Veterans of Foreign Wars (VFW)
	Asia Institute Crane House
	Kentucky Harm Reduction Coalition
	Moore Temple
	St Mary's Center
	St. Peter's United Church of Christ
	Beulah Presbyterian Church
	Healthy West Louisville
	SVDP Location Replacing Goodwill Encampment due to Weather Forecast
	Dismas Charities
	New Legacy
	American Turkish Friendship Association
	Second Presbyterian Church
	Catholic Charities of Louisville Sister Visitor Center
	Immaculate Heart of Mary Catholic Church
	Fountain of Life Ministry Center
	YouthBuild Louisville
	University of Louisville Early Learning Center
	Grace Hope Presbyterian Church
	Bridgehaven Mental Health Services
	Beechmont Baptist Church
	St. Vincent de Paul Louisville
	Molo Village CDC
	Mt Olive Missionary Baptist Church
	Family Health Centers - Portland
	South Louisville Community Ministries
	Queer Kentucky
	Elderserve INC
	The Black Counseling and Consulting Collective
	Play Cousins Collective
	Lynnhurst United Church of Christ
	Seven Counties Services
	Franciscan Kitchen
	Goodwill Opportunity Center
	Louisville New Life Seventh-day Adventist Church
	St George Community Center
	Salvation Army
	Beacon House

Assigned Organization Type	Hosting Organization Name
	Fourth Avenue United Methodist Church
	Interfaith Paths to Peace
	Guiding Light Islamic Center
	MELANnaire Marketplace
	La Casita Center
Senior Care Service Provider	St. Columba Court Apartments
	Stephen Foster Senior Living
	St. Williams Apartments
	Jencare Senior Medical Center
	BUENA VIDA ADHC
	Regis Wood Nursing Home
	St. John Gardens Apartments
	Beehive Homes
	California Square
	The Grand Senior Living
	J.O. Blanton House
	Clifton Oaks Care Center
Youth Service Provider	Robbie Valentine Summer Basketball Camp

Note: Organization classifications were based on the organization type recorded in programmatic records and, when needed, the organization's self-described primary function. Each organization was assigned to one mutually exclusive category. Categories reflect organizational type rather than the specific services provided at individual event locations.

Table S2. Original Hosting Organization Categories and Recoding into the Analytic Categories

Original category	Original frequency	Analytic category
Business organization	90	Business organization
Non-profit organization	242	Non-profit organization
Governmental agency	142	Government agency
Medical service provider	53	Medical service provider
Mental health service provider	6	Medical service provider
Festival	3	Other service provider
Homeless encampment site	5	Other service provider
Independent organization	9	Other service provider
Inmate transitional service provider	2	Other service provider
Senior care service provider	20	Other service provider
Youth service provider	3	Other service provider
**Total**	**575**	

Note: After recoding, the 575 events with recorded hosting organization information comprised 90 business-organization events, 242 non-profit-organization events, 142 government-agency events, 59 medical-service-provider events, and 42 other-service-provider events.

Table S3. Collaborators that partnered with LMPHW for Vaccine Events

	Events (n)	Percent
Advanced Practice Registered Nurse Volunteer	13.0	2.2
Baptist Health	4.0	0.7
Bluewater Diagnostic Laboratory	35.0	5.9
Family Health Centers - Phoenix	4.0	0.7
Kentucky Nurses Association	274.0	46.0
National Guard	103.0	17.3
Norton Healthcare	9.0	1.5
NuLease Medical Solutions	43.0	7.2
Louisville Metro Police Department	35.0	5.9
Park Duvalle Community Health Centers	22.0	3.7
University of Louisville Health	7.0	1.2
Wild Health	11.0	1.8
Unknown	36.0	6.0
Total	596.0	100.0

Partner counts exceed the number of partnered events because some events involved more than one collaborating organization. Percentages are based on partner-event records rather than unique events.

Table S4. Event Characteristics and COVID-19 Vaccine Doses Administered per Event During LMPHW Vaccine Campaign — Jefferson County, KY, March 2021–May 2022

Variable	Unadjusted b (95% CI)	P value	Adjusted b (95% CI)	P value
Duration of Event (per hour)	4.4 (1.9, 6.8)	<0.001	14.9 (11.5, 18.4)	<0.001
Event Time (ref: Afternoon)				
Morning	-37.8 (-57.4, -18.1)	<0.001	-6.3 (-23.9, 11.4)	0.487
Noon	-29.5 (-42.3, -16.8)	<0.001	-7.6 (-19.1, 3.9)	0.195
Evening	-20.6 (-34.1, -7.1)	0.003	-13.3 (-25.9, -0.7)	0.039
Weekend (ref: Weekday)	51.7 (38.2, 65.2)	<0.001	45.7 (32.3, 59.1)	<0.001
Season (ref: Spring 2021)				
Summer 2021	-18.4 (-33.3, -3.4)	0.016	-24.0 (-37.1, -11.0)	<0.001
Fall 2021	-11.2 (-26.3, 3.9)	0.144	-17.1 (-30.8, -3.3)	0.015
Winter 2021–2022	26.3 (8.3, 44.4)	0.004	28.4 (12.2, 44.7)	0.001
Spring 2022	37.4 (16.9, 57.9)	<0.001	40.5 (22.7, 58.2)	<0.001
Event Hosting Organization Type (ref: Government Agency)				
Non-Profit Organization	-3.7 (-17.2, 9.9)	0.595	18.4 (4.4, 32.5)	0.001
Business Organization	-17.1 (-34.5, 0.3)	0.055	23.8 (7.8, 39.9)	0.004
Medical Service Provider	-25.0 (-44.9, -5.0)	0.014	20.8 (2.4, 39.3)	0.027
Other Service Provider	-35.7 (-57.7, -13.7)	0.002	4.3 (-15.6, 24.3)	0.669
Unknown	-36.2 (-53.4, -18.9)	<0.001	-79.3 (-98.8, -59.9)	<0.001
Event Partnership Status (ref: Partnered Event)				
LMPHW-only event	50.6 (34.6, 66.7)	<0.001	46.2 (30.1, 62.2)	<0.001

Note: b = unstandardized regression coefficient; CI = confidence interval. Estimates are from unadjusted and adjusted linear regression models of COVID-19 vaccine doses administered per event. Sample sizes varied slightly across unadjusted models because of missing covariate data. The adjusted model included 630 events with non-missing dose counts and complete data on all model covariates. Unknown refers to events with no recorded hosting organization. LMPHW-only events were conducted independently by the Louisville Metro Department of Public Health and Wellness without formal external partners. Reference categories are indicated in parentheses.

Table S5. Negative Binomial Sensitivity Analysis of Event Characteristics and COVID-19 Vaccine Doses Administered per Event During the LMPHW Vaccination Campaign, Jefferson County, Kentucky, March 2021–May 2022

**Variable**	**Adjusted IRR (95% CI)**	**P value**
Duration of Event (per hour)	1.22 (1.16, 1.29)	<0.001
Event Time (ref: Afternoon)		
Morning	0.71 (0.53, 0.97)	0.029
Noon	0.91 (0.75, 1.11)	0.364
Evening	0.91 (0.73, 1.12)	0.371
Weekend (ref: Weekday)	2.17 (1.73, 2.72)	<0.001
Season (ref: Spring 2021)		
Summer 2021	0.59 (0.48, 0.74)	<0.001
Fall 2021	0.76 (0.61, 0.96)	0.021
Winter 2021–2022	1.16 (0.89, 1.51)	0.283
Spring 2022	2.03 (1.50, 2.74)	<0.001
Event Hosting Organization Type (ref: Government Agency)		
Non-Profit Organization	1.38 (1.09, 1.75)	0.008
Business Organization	1.74 (1.33, 2.27)	<0.001
Medical Service Provider	1.59 (1.16, 2.17)	0.004
Other Service Provider	0.85 (0.60, 1.20)	0.361
Unknown	0.32 (0.24, 0.44)	<0.001
Event Partnership Status (ref: Partnered Event)		
LMPHW-only event	2.47 (1.87, 3.25)	<0.001

Note: IRR = incidence rate ratio; CI = confidence interval. Estimates are from an adjusted negative binomial regression model using the untransformed number of COVID-19 vaccine doses administered per event. The model included 630 events with complete covariate data. Unknown refers to events with no recorded hosting organization. LMPHW-only events were conducted independently by the Louisville Metro Department of Public Health and Wellness without formal external partners. Reference categories are indicated in parentheses.

Table S6. Comparison of Included Events and Events Excluded Because of Missing Dose Counts

Characteristic	Included events, n = 631	Excluded events, n = 31
Event duration, hours		
Mean (SD)	3.6 (2.1)	2.8 (1.2)
Median (IQR)	3.0 (2.0–4.0)	2.3 (2.0–3.3)
Duration available, n	630	28
Event time, n (%)		
Morning	45 (7.1)	2 (6.5)
Noon	131 (20.8)	9 (29.0)
Afternoon	343 (54.4)	14 (45.2)
Evening	112 (17.8)	3 (9.7)
Missing	0 (0.0)	3 (9.7)
Day of Week, n (%)		
Weekday	536 (84.9)	27 (87.1)
Weekend	95 (15.1)	4 (12.9)
Season, n (%)		
Spring 2021	99 (15.7)	0 (0.0)
Summer 2021	203 (32.2)	2 (6.5)
Fall 2021	189 (30.0)	5 (16.1)
Winter 2021–2022	85 (13.5)	21 (67.7)
Spring 2022	55 (8.7)	3 (9.7)
Hosting Organization Type, n (%)		
Non-profit organization	233 (36.9)	9 (29.0)
Government agency	133 (21.1)	9 (29.0)
Business organization	82 (13.0)	8 (25.8)
Medical service provider	55 (8.7)	4 (12.9)
Other service provider	42 (6.7)	0 (0.0)
Unknown	86 (13.6)	1 (3.2)
Partnership Status, n (%)		
Partnered event	565 (89.5)	31 (100.0)
LMPHW-only event	66 (10.5)	0 (0.0)

Note: Included events had recorded dose counts; excluded events were missing dose counts. Percentages are calculated within inclusion group. Event duration was available for 630 included events and 28 excluded events. Event-time information was missing for three excluded events. SD = standard deviation; IQR = interquartile range; LMPHW = Louisville Metro Department of Public Health and Wellness.

Figure S1. Monthly COVID-19 Vaccine Doses Administered and Events by Partnership Status — LMPHW Vaccine Campaign, Jefferson County, KY, Mar 2021–May 2022

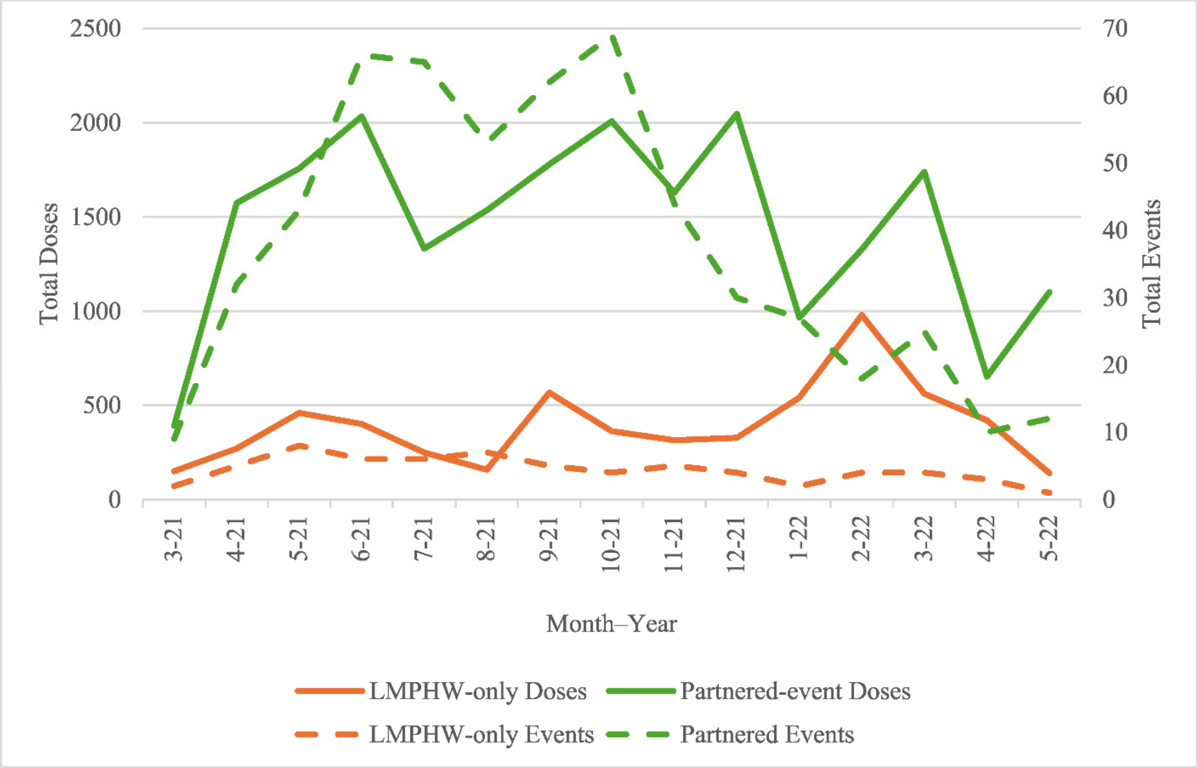

Note. Monthly vaccine doses administered (solid lines; left y-axis) and number of events (dashed lines; right y-axis) by partnership status, Louisville Metro Department of Public Health and Wellness (LMPHW) vaccine campaign—Jefferson County, Kentucky, March 2021–May 2022. Doses reflect events with non-missing dose counts; event counts include all scheduled events.

Figure S2. Monthly COVID-19 Vaccine Doses Administered and Events by Day of Week — LMPHW Vaccine Campaign, Jefferson County, KY, Mar 2021–May 2022

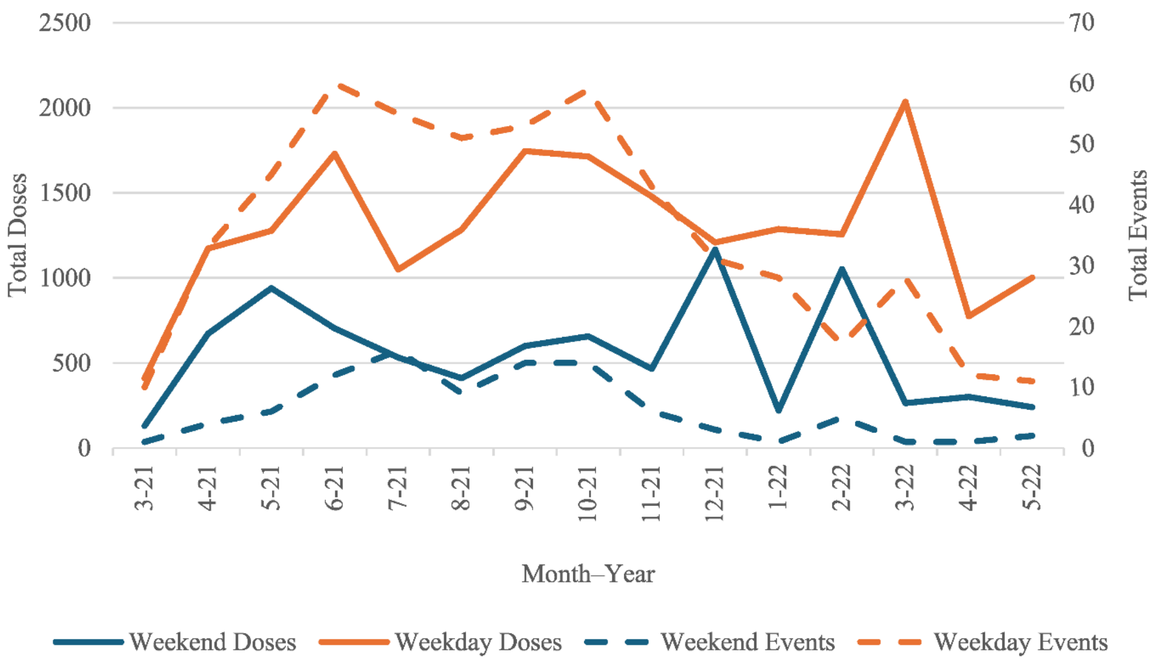

Note. Monthly vaccine doses administered (solid lines; left y-axis) and number of events (dashed lines; right y-axis) by day of week, LMPHW vaccine campaign—Jefferson County, Kentucky, March 2021–May 2022. Doses reflect events with non-missing dose counts; event counts include all scheduled events.
